# Epigenetic regulation of brain development, plasticity, and response to early-life stress

**DOI:** 10.1038/s41386-025-02179-z

**Published:** 2025-08-06

**Authors:** Catherine Jensen Peña

**Affiliations:** Princeton Neuroscience Institute, Princeton University, Princeton, NJ USA

**Keywords:** Neuronal development, Epigenetics and behaviour

## Abstract

Brain development is choreographed by complex gene programs, regulated in turn by epigenetic mechanisms. Far from being complete at birth, both the brain and epigenome continue to mature postnatally. Recent research has found postnatal maturation of the epigenome—including cell-type specific patterns of DNA methylation, chromatin modifications, and non-coding RNAs—to be largely complete by the peri-adolescent period. However, a feature of neurons is their plasticity and dynamic responsiveness to environmental and other signals, and epigenetic mechanisms help govern both critical period and life-long plasticity. Environmental perturbations during development, such as early-life stress, can also become encoded in the epigenome. Evidence from human and non-human animal studies of early-life stress has converged on long-lasting epigenetic changes at several key genes which confer functional changes in stress response, as well as epigenome-wide changes including accelerated epigenetic aging. This review describes epigenetic processes and synthesizes recent literature on postnatal epigenome maturation, the relationship between the epigenome and postnatal sensitive periods and plasticity, and the impact of early-life stress on epigenetic development.

## Introduction

Brain development is orchestrated by complex molecular gradients and signaling pathways that give rise to regional patterning, a diversity of cell types, maturation of cells, and circuit formation. These coordinated changes in gene expression are regulated by the epigenome [1–4]. Cell-type-specific maturation of the epigenome extends into the postnatal period, where it continues to support cellular maturation, circuit refinement, and regulates the pace of critical period plasticity. Proper maturation of these epigenetic mechanisms is essential for healthy neurodevelopment.

The epigenome is thought to sit at the interface between brain development and the environment, allowing the epigenome to fine-tune gene expression in response to physiological needs. Protracted postnatal maturation of the epigenome may leave these developmental processes particularly vulnerable to early-life stress (ELS) and other perturbations. ELS has been shown to exert long-lasting changes in transcription across the genome in multiple brain regions [5–8], implicating persistent changes in epigenetic regulation. Two decades of research now shows that ELS alters genes that regulate stress response, neural plasticity, and epigenetic function itself [9, 10]. These changes in the epigenome are thought to contribute to long-lasting functional changes in the brain, heightened stress sensitivity, and vulnerability to neuropsychiatric disorders. Understanding both normal epigenetic development—and how ELS alters maturation of the epigenome—is critical for identifying mechanisms of risk and resilience and for developing targeted interventions.

## What is “epigenetics?”

The term “epigenetics” was coined by Conrad Waddington in 1942 as a way to explain the causal mechanisms of how genotype leads to phenotype in the context of development and environment (both a cell’s environment, and the organism’s environment) through refined control of gene activity [11]. Waddington’s concept of epigenetics linked epigenesis, preformation, and genetics. However, much like DNA across generations, the concept has been recombined and mutated over the years [12] such that the most popular definition has come to refer to factors that alter gene expression without altering underlying DNA sequence, associating “epi” with “above the genome” rather than epigenesis. Here, “epigenetics” is defined as persistent changes in transcriptional state or transcriptional potential, regulated by molecular mechanisms beyond DNA sequence, to influence phenotype.

**Fig. 1:**
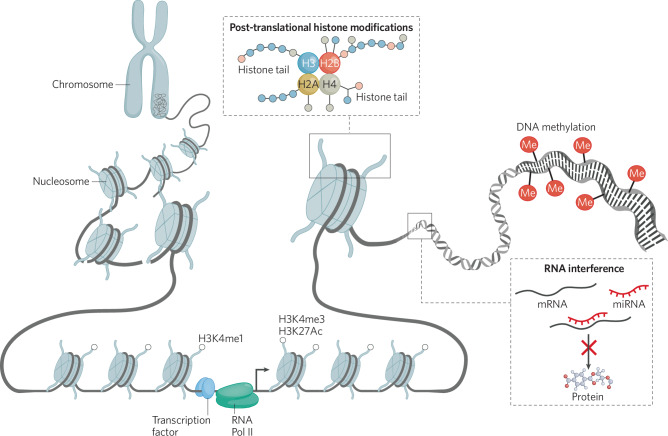
Epigenetic modifications. Nucleosomes of chromatin consist of DNA wrapped around histone proteins. Post-translational modifications to histone proteins (methylation, acetylation, etc) and DNA (methylation) influence compaction of chromatin and recruitment or ejection of chromatin remodeling complexes, methyl binding proteins, transcription factors, and RNA polymerase to fine-tine gene expression. Non-coding RNAs such as microRNAs can also interfere with translation of mRNAs into protein.

We now associate the concept of epigenetics with physical modifications and processes that regulate gene expression including DNA methylation, post-translational histone modifications (PTHMs), and regulation of mRNA by non-coding RNAs (Fig. 1). While it was once thought that some epigenetic modifications were more stable or dynamic than others [1, 13], it is now appreciated that DNA methylation and PTHMs can be rapidly added or removed, but can also be relatively stable across cell division or an organism’s lifespan, depending on age, gene in question, and environmental factors. DNA methylation, PTHMs, and non-coding RNAs can also influence each other [14, 15]. Our ultimate interest in these modifications is in their ability to influence current or future gene expression (and physiological processes of the cell). Simple presence of a modification by itself does not necessarily equate to functional change. Finally, it is important to keep in mind that epigenetic modifications do not necessarily toggle gene expression between ‘off’ and ‘on’ positions, but act more like a volume dial to fine-tune when and how much genes are transcribed.

### DNA methylation

DNA methylation refers to covalent addition of a methyl group to DNA, which predominately occurs at the 5′ carbon of cytosine bases (5mC) followed by guanine bases (CpG dinucleotides) in mammals. DNA methylation is catalyzed by DNA methyltransferases [16]. The methyltransferase DNMT3a and DNMT3b establishes de novo methylation by adding methyl groups to previously unmethylated cytosines. In contrast, DNMT1 has a higher affinity for hemi-methylated DNA and predominately participates in maintenance methylation during cell division, such that the pattern of methylation present in the parent cell is faithfully copied to daughter cells. DNA can also be actively demethylated by ten-eleven translocation (TET) enzymes which catalyze hydroxylation of 5mC to 5-hydroxymethylcytosine (5hmC) and then through additional oxidized states which can then be replaced by unmethylated cytosine through base excision repair [17]. In addition to these enzymatic “writers” and “erasers,” DNA methyl binding “reader” proteins such as MeCP2 and methyl-CpG binding domain proteins MBD1-MBD4 recognize methylated DNA and recruit effectors including histone deacetylases (HDACs, described below) [13].

Methylation patterns vary across development and across the genome. After fertilization, there are two waves of demethylation and re-methylation of the entire genome. First, the paternal genome is rapidly and actively demethylated, followed by slower passive demethylation of the maternal genome [18, 19]. At the blastocyst stage, the genome is remethylated [20]. In mature cells, promoters and first exons of expressed genes are largely unmethylated, while transposon-derived sequences and imprinted genes are densely methylated, and other genomic regions have variable methylation [19]. Methylation of transposable elements and imprinted genes serves to silence them and protect genomic stability. Methylation in gene enhancers, promoters, and first exons also represses their expression. However, methylation in other exons and introns can increase gene expression and influence alternative splicing [21, 22]. In contrast, 5hmC is associated with un-silencing of genes and active gene expression [23].

DNA methylation is stable through cell division, and contributes to the general stability of gene expression states across a cell’s life [13]. While this is especially true in silenced regions of the genome, we now appreciate that DNA methylation can also be highly dynamic, particularly in the brain. Neuronal activity promotes rapid changes in DNA methylation, including both de novo methylation and TET1-mediated demethylation, particularly at immediate-early genes and genes that regulate neuronal plasticity [24–26].

### Chromatin modifications

Chromatin consists of DNA is wrapped around histone proteins and helps keep DNA compacted and organized in the nucleus. In a single nucleosome, the unit of chromatin, 147 bases of DNA are wrapped around a histone octamer which consists of two copies each of histone proteins H2A, H2B, H3, and H4 [27]. The DNA stretching between nucleosomes is referred to as linker DNA and can be variable in length. Indeed, nucleosomes undergo remodeling in an ATP-dependent manner and DNA slides around histone octamers like thread off a spool to open chromatin and make DNA accessible to RNA polymerase for gene transcription. Each histone protein has an N-terminal tail that protrudes from the nucleosome and can be post-translationally modified by addition of acetylation, methylation, phosphorylation, ubiquitination, and even dopaminylation and serotonylation [28, 29]. Chromatin modifications are “written” by enzymes including families of histone methyltransferases [HMTs, including lysine methyltransferases (KMTs) and arginine methyltransferases(PRMTs)], histone acetyltransferases (HATs), kinases, and transglutaminases that add methyl, acetyl, phosphoryl, or monoamine groups to histones, respectively [27, 29]. Modifications are removed or “erased” by lysine demethylases (KDMs), histone deacetylases (HDACs), and phosphatases.

Each histone tail can be post-translationally modified at dozens of amino acid residues independently (and in the case of lysine methylation, can be mono-, di-, or tri-methylated), giving rise to a “histone code” with enormous combinatorial complexity [30]. For example: histone H3 has 13 lysines that can have 0, 1, 2, or 3 methyl groups; if all modifications were independent and equally likely (which is not the case) there would be more than 67 million (4^13) patterns of lysine methylation on H3 alone in each nucleosome, in one cell. These PTHMs—alone or in combination—are associated with compacted and silenced chromatin, loosely wound and accessible chromatin permissive for gene expression, alternative splicing of genes, primed and poised chromatin that facilitates stimulus-dependent gene expression, or active gene expression. Chromatin modifications are therefore considered to be dynamic regulators of gene activity. Acetyl and phosphoryl groups are both negatively charged and neutralize the positive charge of histone proteins, thereby decreasing chromatin compaction and decreasing the electrostatic affinity of negatively-charged DNA to the nucleosome [30]. Because of this, histone acetylation is generally associated with a more open chromatin conformation and active gene expression. Methylation of histone lysine and arginine residues, on the other hand, can be associated with active transcription, repression, or other states, depending on the location (residue) and degree (mono-, di-, or tri-) of methylation. For example, histone-3 lysine-4 monomethylation (H3K4me1) is associated with primed or active enhancers, while trimethylation at the same location (H3K4me3) is associated with active gene expression, and methylation at histone-3 lysine-9 (H3K9) or histone-3 lysine-27 (H3K27) is associated with gene repression. Histone “marks” also occur in combination, with distinct function. For example, enhancers marked by both permissive H3K4me1 and repressive H3K27me3 are “poised” to become active (by removal of repressive complexes) upon stimulation [31]. Changes in chromatin state can occur rapidly in response to simulation (such as cellular differentiation or neural activity) [32, 33], but can also be relatively stable across the lifespan [34]. Histone variants and chromatin remodeling also regulate gene expression and are covered in detail in previous issues [27].

### Non-coding RNAs

Non-coding RNAs are also considered epigenetic regulators of gene activity, as they can effect transcriptional repression, splicing, sequestration, and degradation of target genes [35]. There are many types of non-coding RNAs, predominately classified by their size, although in neurodevelopment and neuropsychiatric disease the most attention has been paid to micro-RNAs (miRNAs) and long noncoding RNAs (lncRNAs).

miRNAs range in size from 19 to 24 bp and are encoded at widespread locations throughout the genome. After processing, miRNAs base-pair with complementary sequences of mRNAs in a RNA-induced Silencing Complex (RISC) which silences mRNA by sequestration, degradation/cleavage, or translational suppression. miRNAs have varying degrees of specificity, with some able to act on hundreds of genes simultaneously. miRNAs can be brain-enriched [36], neural activity-dependent [37], localized to dendrites [38] to control dendritic development [39], and involved in neurological and neuropsychiatric diseases [35, 40].

lncRNAs are greater than 200 base pairs but can be many kilobases long (e.g., *Xist*, which is 17Kb in humans). They are encoded at widespread locations throughout the genome. Some lncRNAs are even more highly expressed than constitutive cell-homeostasis genes. lncRNAs and bind complementary single-stranded DNA or double-stranded DNA and are involved in X-chromosome inactivation, genomic imprinting, gene *activation* (e.g., enhancer RNAs), and scaffolding of 3-D chromatin loops [41]. Like other non-coding RNAs, lncRNAs have spatial, temporal, cell-type-specific, and sub-cellular specific expression patterns, suggesting transcription is tightly regulated [35], and have been implicated in psychiatric disease [42, 43].

## Postnatal maturation and plasticity of the epigenome

Epigenetic regulation is essential to embryological development—and indeed was conceptualized to help explain the “bifurcating creodes” of early cell fate lineage commitment—but maturation of the epigenome continues postnatally in post-mitotic cells in a brain region and cell-type-specific manner. These changes in the epigenome reflect maturation of the cells themselves. After differentiation, neurons must migrate towards their final location in the brain, stop migration, grow axonal and dendritic branches, form synapses, generate activity-dependent neurotransmitter release, etc. Each of these maturational stages require different molecular programs, which are stably turned on and off by changes in the epigenome.

**Fig. 2:**
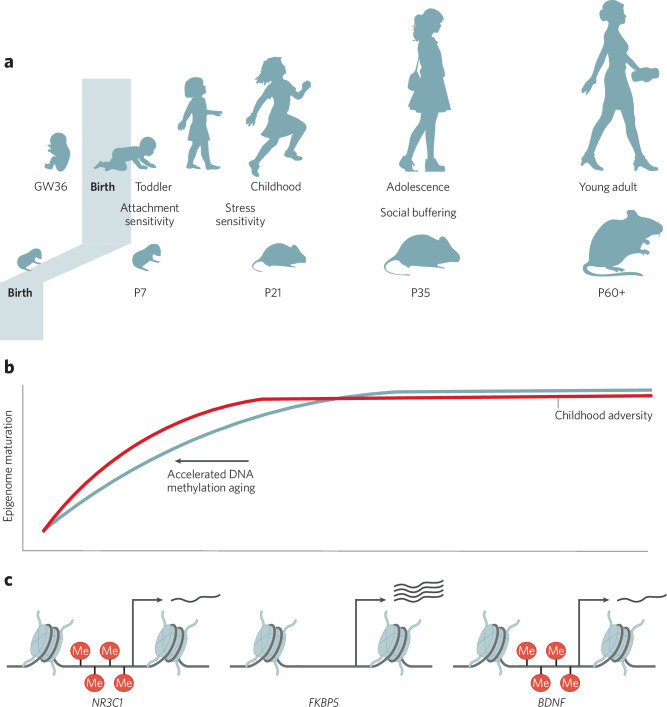
Early-life stress influences the developing epigenome. **a** Postnatal development among humans and rodents is marked by periods of attachment and stress sensitivity, the negative impact of which may be buffered by positive social environments. **b** The neural epigenome matures in a cell-type-specific manner until ~adolescence at which point it is relatively stable. Early-life stress accelerates aging of the DNA methylome. **c** Early-life stress alters DNA methylation at *NR3C1*, *FKBP5*, and *BDNF* in human and rodent studies. Increased or decreased promoter methylation is associated with suppressed or augmented gene expression, respectively.

Significant maturation of cell-type-specific DNA methylation and chromatin modification patterns occurs between postnatal weeks 1–3 in mice, with little further maturation of the epigenome after three weeks of age to eight weeks or in aged mice [34, 44]. For example, enhancers within somatostatin neurons that are active in adulthood have a gradual accumulation of H3K27Ac (which marks active enhancers) until postnatal week 3, and enhancers that are suppressed in those neurons have a gradual depletion of H3K27Ac over the same period [34]. Similarly, examining brain region-specific expression of DNMTs across postnatal development reveals a decline in DNMT1 from birth to postnatal day P21, after which levels are stable, and a postnatal peak in DNMT3a levels in hippocampus, amygdala, and striatum between P4-10 which also declines until P21 with stable levels thereafter [45]. In human cortex, DNA methylation levels change across postnatal development in about 2.4% of genomic sites probed, with both increases or decreases in methylation identified at different sites, and with postnatal changes in DNA methylation largely occurring in neurons and not non-neurons [46]. For genomic regions that show DNA methylation changes across development, change occurs at a rate of about 0.1% per year [46]. Several genome-wide studies have now shown that while CpG methylation is largely stable from birth to adulthood in both mouse and human cortex and hippocampus, there is a dramatic postnatal accumulation of non-CG methylation, peaking in adolescence when the brain is highly plastic, and largely in neurons [47, 48]. Chromatin accessibility profiling in the developing human cortex from fetal through adult ages also shows postnatal cell-type-specific maturation of chromatin, which is complete for most cortical cell types around adolescence — here with a notable exception of oligodendrocytes whose chromatin continues to mature to adulthood [49]. We may therefore consider the neural epigenome fully mature by three weeks of age in mice and during adolescence in humans (Fig. 2a, b). However, this also leaves open the possibility that postnatal insults which alter the epigenome during this period of maturation may become “crystalized” and persist across the lifespan (see Section 3).

### Epigenetic regulation of sensitive periods in postnatal development

Sensitive periods are epochs of life when experience-dependent plasticity is enhanced relative to periods before or after the window; when a lack of expected input results in a failure of brain maturation or learning in some domain (classically, patterning of the visual cortex for binocular vision, or language learning) that can no longer be fully recovered, that window is classified as a critical period [50, 51]. As elegantly put by Piekarski and colleagues: “Postnatal brain development is studded with sensitive periods” [52]. The epigenome has postnatal sensitive periods, and may indeed help regulate critical period timing.

DNA methylation and one of its prominent binding proteins, MeCP2, have been best studied as critical period regulators. Visual circuitry has been used as a “gold standard” to study critical periods given its conservation across species, relatively late critical period in rodents from P26-P32 (rodent eye opening doesn’t occur until ~P13 in mice), well-defined outcome measures, and ease of manipulating sensory input. In primary visual cortex (V1), MeCP2 increases during the critical period, particularly in glutamatergic neurons [53]. Mice lacking *Mecp2* have a critical period that is advanced by about 10 days, including advanced maturation of parvalbumin interneurons and perineuronal nets — structures associated with critical period maturation [53, 54]. Similar (albeit less pronounced) critical period advancement has also been detected in somatosensory cortex of *Mecp2*-null mice [55]. It has been proposed that accumulation of methylation across postnatal development (perhaps largely at non-CG sites, as described above) serves generally to close critical periods, as pharmacological inhibition of DNA methylation in adult animals (which should release transcriptional repression at gene promoters) can restore critical-period like plasticity in cortical brain regions [56]. The ability for the brain to establish, read out, and respond to DNA methylation properly is therefore essential for properly timing critical period plasticity and brain development more broadly.

Chromatin profiles support the hypothesis that critical period plasticity requires enhanced ability to actively transcribe genes. Clever study design took advantage of the fact that among songbirds, the window for juvenile males to learn from a song tutor is extended if males are reared in isolation and never hear a tutor, and thus differences in epigenetic state of the auditory cortex can be measured in birds of the same age but different plasticity states. Isolated birds in an extended plasticity state have hundreds more genes associated with H3K4me2 and RNA POL2, markers of active gene expression, while tutored birds whose critical periods have closed have hundreds more genes associated H3K27me3, a marker of transcriptional suppression, and the genes that differ in expression are themselves related to epigenetic regulation [57]. Indeed, histone deacetylase inhibition (HDACi, which serves to maintain high levels of histone acetylation, which is associated with a more open and permissive transcriptional state) has been found to enhance plasticity across brain systems and in both humans and non-human animal models [51, 58, 59]. Intriguingly, environmental enrichment also enriches histone acetylation levels and may therefore be used as a natural method to restore or enhance brain plasticity [51, 60].

MicroRNAs also govern aspects of critical period plasticity. More than 2000 miRNAs were found to change expression across postnatal development of mouse visual cortex [61]. Among these, miR-29a is upregulated across postnatal development, peaking around P60 at 30-fold higher levels than at P10 (while in contrast, *Dnmt3a* expression in V1 falls from P10 to P25 and remains stable until at least P200) [62]. miR-29a regulates neuronal maturation and its predicted gene targets include genes related to extracellular matrix remodeling enzymes and epigenetic factors [62]. Early over-expression of miR-29a in V1 suppressed *Dnmt3a* expression, advanced maturation of perineuronal nets and suppressed plasticity, while inhibition of miR-29a in adulthood elevated *Dnmt3a* and re-opened plasticity akin to the juvenile critical period [62]. miRNAs therefore collaborate with other epigenome modifying enzymes, including DNMTs, to regulate critical period plasticity through functional changes in brain maturation.

### Epigenetic regulation of hormonal sensitive periods

Hormones also regulate stereotyped critical periods in the brain and interact directly with the epigenome. Masculinization of the brain occurs during a critical period around birth in response to a surge in testosterone (or its metabolite estradiol) in both primates and rodents with a Y chromosome [63]. In rodents, delaying this testosterone surge even by only a few days prevents masculinization of the brain and emergence of male-typical reproductive behaviors in response to testosterone later in life [64]. Because the brain has female-typical patterning in the absence of testosterone or estradiol, “feminization” of the brain was long considered the “default” state. These steroid hormones bind to their receptors, which act as transcription factors in the nucleus and can directly regulate gene expression as well as orchestrate epigone remodeling [65]. Research in the last decade has shown that long-lasting molecular changes associated with both male- and female-typical patterning are actively maintained by epigenetic mechanisms, including DNA methylation. One primary effect of the neonatal steroid hormone surge is to actively suppress DNMT activity in the medial preoptic area of the hypothalamus (a sexually dimorphic brain region), which in turn reduces DNA methylation and allows expression of genes that typically have higher expression in males [66]. Therefore, rather than a “default” female-typical state of the brain, a female-typical profile of gene expression needs to be actively maintained across the lifespan by DNA methylation [66].

Puberty marks a period of dramatic neuroendocrine transition and reorganization, a period of increased risk for psychiatric disease, and another sensitive period for the epigenome. Looking only at salivary DNA methylation across a ~two-year window spanning onset of puberty, methylation at 2602 sites were found to change with puberty (on an Illumina EPIC array with 794,811 sites, or 0.33% of sites probed) [67]. More than five-times as many sites had altered methylation in females compared to males across the pubertal transition [67], mirroring the increased risk for psychiatric disease among women at periods of hormonal change [68]. The timing of puberty itself is in fact regulated by epigenetic mechanisms. Initiation of puberty requires release of gonadotropin-releasing hormone (GnRH), which is stimulated by kisspeptin acting vis it’s receptor (KISS1R/GPR54), among other changes. DNA methylation at *Gnrh* in the hypothalamus falls at puberty in non-human primates, which allows increased gene expression [69]. In female rats, hypothalamic *Kiss1* gene expression is repressed by DNA methylation and binding of polycomb repressive complexes at the promoter prior to puberty, and initiation of puberty requires epigenetic silencing of two key polycomb group genes to de-repress *Kiss1* [70]. Pharmacological inhibition of DNA methylation delays puberty [70]. Furthermore, demethylation of the *Kiss1* promoter and increases in H3K4me3 and H3K9-14Ac—chromatin modifications associated with active gene expression—are also required for puberty onset [70]. Additional epigenetic changes associated with puberty are thoroughly described by Morrison and colleagues, including sex-specific changes in histone deacetylases and microRNAs [71]. These epigenomic changes during puberty and adolescence poise the brain to respond to its environment and mark a period of heightened plasticity, as well as heightened sensitivity to stress, substances, and other perturbations.

These examples interactions between hormones and the epigenome are not exhaustive. For more comprehensive reading, see [63, 71, 72].

### Lifelong plasticity of the epigenome

While a majority of gene programs are stable by adulthood, a feature of neurons is their plasticity and dynamic responsiveness to environmental and other signals. The epigenome supports lifelong synaptic plasticity [27, 73, 74]. Neuronal activity requires and results in molecular changes [75]. Upon depolarization, immediate-early genes (IEGs, such as *Fos* and *Arc*) are transcribed within minutes [76, 77], with additional “late response” genes transcribed within hours [33, 78]. These expression changes are necessary for synaptic remodeling, among other functions [75]. These changes in expression are also accompanied by —and indeed dependent upon— changes in DNA methylation and chromatin state. For example, breakthrough work from more than twenty years ago discovered that depolarization of neurons in vitro induces DNA *de*methylation at the brain-derived neurotrophic factor (*Bdnf*) exon IV promoter and ejection of both the DNA methylation binding protein MeCP2 and repressive chromatin complexes, and that these changes are required for activity-dependent *Bdnf* expression [24]. We now know that chromatin remodeling and changes in DNA 5mC and 5hmC (including both methylation and demethylation) take place within hours of neuronal depolarization across the genome [25]. If neurons were previously quiescent, significant chromatin remodeling also occurs within 1–4 h after stimulation to support early transcriptional response [32, 77, 79], although neurons in a more naturalistic state may already have open promoters and enhancers from continual use [33]. A majority of these activity-dependent changes are to open chromatin and make enhancers and promoters more accessible to transcriptional machinery to facilitate transcription [33, 77, 80]. Such chromatin remodeling is ATP-dependent and relies of the BAF complex to help evict histone octamers, as well as histone acetyl transferases such as CBP (CREB Binding Protein) to acetylate H3K27 on nearby nucleosomes [75]. However, activity-regulated gene expression must also be returned to baseline, which is accomplished by recruitment of repressive nucleosome remodeling complexes and HDACs to gene promoters within hours [80]. These activity-regulated genes govern synaptic transmission, synapse formation, cell-cell signaling, and potassium channel activity across the lifespan [77]. Neuronal activity thus shapes epigenetic regulation of genes, which in turn shapes neuronal activity and synapse and circuit formation.

Therefore, the epigenome can be both stable and dynamic, depending on gene and genomic region, developmental state, and environment.

## Influence of early-life stress on epigenetic development and gene regulation

While stress at any age can be detrimental, stress experienced during development has the potential to alter development itself, including development of the epigenome [81]. Childhood adversity or early-life stress (ELS) can come in many forms, including maltreatment and neglect, poverty, exposure to household or community violence, racism, loss of a parent or caregiver, or loss of one’s home [82–86]. At its broadest definitions, more than half of children are estimated to experience at least one form of ELS, and ELS contributes to 30–40% of all mood, drug, and psychiatric disorders [83, 87, 88]. The contribution of ELS to mood disorder risk eclipses genetic contributions and instead points to a role for the epigenome to mediate long-term changes in gene expression, brain function, and disease susceptibility. Robust literature from human, non-human primate, and rodent studies now demonstrates the impact of ELS on the epigenome and downstream functional consequences.

For more on rodent models of ELS, potential sensitive periods of ELS in humans and rodents, and the impact of ELS on other circuit and molecular mechanisms, see [81] and elsewhere in this issue [89].

### Timing of adversity on the epigenome

Different brain systems mature at different ages and rates, and the timing of stress experience will therefore impact maturation of different neural and physiological processes. Studies have identified effects of different stressors during early childhood, middle childhood, or puberty on different aspects of anxiety and depression [90–95]. One study explicitly designed to test the hypothesis that there may be critical periods of ELS to impact the epigenome found that ELS experienced in very early childhood (ages 0–3) appears to have the largest impact on DNA methylation in peripheral blood leukocytes of 7 year-old children compared to adversity experienced from ages 3–5 or 6–7, although adversity at older ages was not assessed [96]. This effect was true across nearly all types of adversity (with the exception of caregiver physical or emotional abuse, which had the largest impact in middle childhood) [96]. Importantly, this study was able to rule out alternative models, showing that DNA methylation changes were not predicted by stress accumulation or recency of stress. However, a systematic review of the human literature did not converge on any single period of childhood most sensitive to childhood maltreatment on mental health disorders [97]. This is likely due to the contributions of multiple brain systems to different aspects of stress response and psychiatric disease, each affected in different ways by stress at different ages [81, 98–101].

### ELS alters epigenetic regulation of key genes

Decades of research in humans and non-human animal models now confirms that ELS can alter epigenetic regulation of key stress- and plasticity-related genes [10, 102–104]. In particular, there has been widespread focus on the glucocorticoid receptor (GR, encoded by the *NR3C1* gene), the glucocorticoid receptor co-chaperone protein FK506-binding protein 51 (encoded by *FKBP5*), and brain derived neurotrophic factor (encoded by *BDNF*) (Fig. 2c). For a systematic review of the human literature and tables, see [104].

Work in rats in 2004 demonstrating the role of the epigenome in mediating the long-lasting impact of the early environment on hypothalamic-pituitary-adrenal (HPA) axis function and stress response set the field on fire [105]. This early work showed that rats pups that experienced low levels of active maternal care in the first 10 postnatal days had higher levels of DNA methylation around the *Nr3c1* exon 1_7_ promoter (equivalent to the human *NR3C1* 1 _F_ promoter variant) in the hippocampus, and specifically at a CpG site overlapping with a binding site for the transcription factor EGR1/NGFI-A [105]. Lower levels of active maternal care was also associated with lower H3K9 acetylation and lower EGR1/NGFI-A binding at the *Nr3c1* 1_7_ promoter, lower expression of *Nr3c1* mRNA and protein in hippocampus, and reduced HPA-axis negative feedback regulation and greater/prolonged stress response [105]. Moreover, the epigenetic and functional effects of low levels of active maternal care could be mimicked by infusion of the methyl donor L-methionine and reversed by systemic treatment of offspring with the HDAC inhibitor trichostatin A [105, 106]. Cross-fostering (adoption) of pups between low- and high- care dams in these studies also demonstrates the importance of the postnatal rearing environment experienced rather than inherited epigenetic factors in programming offspring stress response. Epigenetic regulation of NR3C1 by stress has since been extended to humans [107–110] and largely supported in meta-analyses, with exceptions [104, 111, 112].

Glucocorticoid receptor activity is modulated by FKBP5, which is also epigenetically regulated by ELS [113]. FKBP5 is a co-chaperone protein that binds GR in the cytoplasm and reduces affinity of GR for cortisol (humans) or corticosterone (rodents), which delays translocation of ligand-bound GR to the nucleus (where GR acts as a transcription factor to regulate thousands of genes across the genome, including *FKBP5*) and alters negative feedback of the HPA axis [113]. *Fkbp5* expression is upregulated by stress and synthetic glucocorticoids in a number of brain regions in rodents [114]. A number of *FKBP5* polymorphisms have been identified which confer differing degrees of stress sensitivity, and upon which ELS selectively interacts to alter DNA methylation [113, 115, 116]. In individuals carrying a risk allele, childhood trauma lowers *FKBP5* DNA methylation, which is associated with increased stress-induced expression [115, 116]. Interestingly, this gene x environment interaction in humans was only found with ELS and not with adult stress. The impact of stress and glucocorticoids during development on *FKBP5* epigenetic regulation and function are largely supported by additional human studies [104, 117], studies in rodents, and studies in human hippocampal cell lines treated with glucocorticoids during proliferation [118].

The neuropeptide BDNF is integral to neuronal development, neuronal survival, experience-dependent dendritic remodeling, learning and memory, and synaptic plasticity [119]. BDNF expression and epigenetic regulation is responsive to stress across the lifespan [120]. It was first found in rodents that ELS persistently decreases mRNA expression of *Bdnf* exons IV and/or IX in prefrontal cortex, ventral tegmental area (VTA), and hippocampus, which is mediated by increased DNA methylation and decreased histone acetylation, and reversed by treatment with a DNA methylation inhibitor or antidepressants [121–123]. Child maltreatment has also been found to decrease plasma BDNF protein and increase methylation at salivary and plasma-derived *BDNF*, which intersects with presentation of psychiatric disease [104, 124–127]. These alterations in *Bdnf* regulation and expression have functional consequences on brain development, synaptic plasticity, and cognition.

Unbiased epigenome-wide screens of the impact of child maltreatment on salivary DNA methylation also found significant alterations at *NR3C1*, *FKBP5*, and *BDNF* as candidate genes, although methylation levels at those genes did not predict depression [128]. However, DNA methylation at other plasticity-related genes was found to be altered by child abuse and mediate depression, including at the NMDA receptor gene *GRIN1* [128], and at *OTX2* [129], a transcription factor involved in neural development and critical period plasticity [130]. In mice, ELS suppresses expression of *Otx2* in the VTA during a late juvenile sensitive period, which is necessary and sufficient to cause enduring stress sensitivity [5]. The impact of ELS on both hippocampal *Nr3c1* and VTA *Otx2* may be mediated by thyroid hormone signaling, which is impaired by ELS in rodents and humans, but rescued by handling in rodents [131–133].

The consistent impact of ELS on epigenetic regulation of *NR3C1*, *FKBP5*, and *BDNF* across human and other animal studies demonstrates the robustness of these findings and their functional consequences on stress response and neuropsychiatric disease susceptibility.

### The impact of ELS at an epigenome-wide level

ELS exerts long-lasting changes in gene expression across the genome, in addition to the candidate genes described above [5–8]. ELS can alter epigenome-modifying enzymes in the brain such as DNA methyltransferases [134–137], histone methyltransferases [138, 139], histone acetyltransferases and histone deacetylases [136, 137, 140, 141], and histone dynamics across the lifespan [139, 142, 143], which suggests epigenetic alterations occur broadly across the genome. Indeed, genome-wide changes in DNA methylation and histone modifications have been identified in blood, salivary, and brain samples from individuals with a history of child maltreatment [104, 128, 144–146]. However, across studies, ELS does not appear to globally increase or decrease DNA methylation but instead shift its deposition patterns. Similarly, ELS in mice shifts cell-type-specific deposition of the histone modification H3K79e2 (a memory marker of past expression which may also influence alternative splicing) [142, 147].

One function of broadly altered chromatin state following ELS may be to prime response to future stress [81]. Epigenetic priming at enhancers facilitates a faster, stronger, or sensitized transcriptional response to a stimulus or exposure, such as stress. Chromatin at primed enhancers is open and accessible to transcriptional machinery and marked by H3K4me1 (while addition of H3K27Ac distinguishes active enhancers) [148, 149]. ELS in mice enriches for H3K4me1 in both VTA and nucleus accumbens (NAc), and in VTA also increases expression of the enzyme that “writes” H3K4me1, *Setd7* [139, 142, 150]. Juvenile (but not adult) overexpression of *Setd7* in NAc is sufficient to mimic ELS and sensitize mice to stress exposure in adulthood [150]. ELS also enriches for H3K27me1 in NAc (specifically in *Drd1*+ neurons) at genes that regulate neuronal excitability [151]. In VTA, juvenile H3K4me1 enrichment via *Setd7* overexpression primes transcription and sensitizes dopamine neuron firing in response to mild adult stress [139]. Together, these studies link epigenomic consequences of ELS to functional circuit-level and behavioral stress sensitivity.

### ELS accelerates epigenetic aging

Epigenetic modifications accumulate slowly across aging within individuals [46]. A study of twins found that monozygotic twins are not only genetically identical, but also nearly *epigenetically* identical in childhood (examining both DNA methylation and histone H3 and H4 acetylation patterns) [152]. However, as twins age, their epigenomes also become more and more distinct, presumably reflecting accumulation of distinct experiences and exposures, such that 50 year-old twins had epigenetic patterns that differed by more than 25% [152]. Accumulation of epigenetic modifications (or an “erosion of the epigenetic landscape”) is also one proposed mechanism of aging itself [153]. “Epigenetic aging,” or the rate of change of DNA methylation and other epigenetic patterns in an individual, may differ from chronological age and is used as a marker of biological aging of different tissues [154, 155]. Different models of epigenetic aging, either across the epigenome or focusing on telomere length specifically (with telomere length inversely related to aging), have been used to attempt to predict overall health and potential longevity [156, 157].

ELS is consistently associated with shorter telomere lengths and accelerated epigenetic aging. Growing up in poor caregiving environments, institutionalization (growing up in a state-run orphanage), unstable family structure, loss of a parent, poorer quality neighborhoods, poverty, childhood maltreatment, and exposure to violence during childhood are all associated with shorter telomere lengths as assayed in children and adults across different populations [158–167]. The impact of ELS on telomere length takes hold by middle childhood [164, 165]. However, various forms of intervention and social support buffer the impact of stress on epigenetic aging. A longitudinal randomized control trial of children in the Bucharest Early Intervention Project found that while institutionalization in early childhood shortened telomeres by middle childhood and adolescence, randomized intervention to high-quality foster care prevented telomere attrition [159, 164]. Similarly, high levels of racism experienced across life among Black men was associated with shorter telomeres, but only among those who had internalized racism such that buffering the psychological impact of racism may also buffer the biological impact [168].

Epigenetic aging, modeled in multiple ways based on DNA methylation across the epigenome (rather than only telomere length), is also accelerated by childhood trauma, neglect, poverty, poor-quality neighborhoods, exposure to violence, peer victimization, and institutionalization (Fig. 2) [169–174]. Girls may be more vulnerable than boys to accelerated epigenetic aging, and there may be sensitive periods for different types of adversity [171]. In a cohort of nearly 1000 blood samples from children sampled from the Avon Longitudinal Study of Parents and Children at age 7.5, epigenetic aging was strongest in girls who experienced sexual, physical, or emotional abuse from approximately 3–5 years old, while boys were most sensitive to financial hardship or neighborhood disadvantage in middle childhood (~7 years old) [171]. However, genome-wide epigenetic aging is also buffered by social support and positive interventions during childhood. For example, while neighborhood disadvantage was associated with accelerated epigenetic aging in three different prediction models, these effects were offset by high levels of neighborhood social cohesion [170].

Accelerated epigenetic aging following ELS is thought to advantageously accelerate brain development to prepare an individual for early independence [175, 176], but also to contribute to the increased risk for both mental health disorders and physical health disorders across tissue types [177]. Just as consistent as these negative findings, however, are findings that interventions, social support, and psychological resilience can buffer the impact of ELS on epigenetic aging, providing hope for those who experienced childhood adversity [178].

## Conclusions and future opportunities for the field

Epigenetic mechanisms, including DNA methylation and chromatin modifications, can confer both stability and dynamic plasticity for developing neural circuits, depending on gene and genomic region, developmental stage, and environment. While research has begun to characterize and appreciate postnatal maturation of the epigenome, this work is still in its infancy. In particular, additional longitudinal studies are needed to characterize cell-type-specific development of chromatin patterns in sub-cortical brain structures, in both humans and rodents. Comparison of developmental milestones across species will provide an essential foundation for translating preclinical studies and informing timing of manipulations and interventions. Additional research on normative brain development can then be used to understand how ELS alters trajectories of development.

Studies from human populations and rodents on the impact of ELS on epigenomic regulation largely support each other, with pre-clinical research effectively testing the causality of mechanisms observed in human samples, and rodent research likewise informing which genes should be investigated further in human studies [105, 129]. Strong evidence has converged on the persistent impact of ELS on epigenetic regulation of key genes governing stress response and plasticity, as well as on accelerated epigenetic aging more broadly [104]. However, additional research harnessing advances in site-specific epigenome editing (i.e., by CRISPR-based catalytically-dead Cas9 tethered to epigenome effectors and targeted to specific DNA sequences) [179] would provide more direct causal evidence for the impact of specific DNA methylation or chromatin changes on neural function, stress response, and behavior.

Encoding of early-life experience in the epigenome represents a biological mechanism for persistent alterations in neural function and neuropsychiatric disease risk. Although rodent studies have found experience-dependent epigenetic alterations to be “druggable” and responsive to pharmacological inhibition of DNA methylation and HDAC inhibitors [56, 105, 106], these drugs are non-specific and unlikely to be desirable treatments to reverse the impact of childhood adversity. However, interventions such as early-life exercise [180] or environmental enrichment [137] may be able to ameliorate the impact of ELS on some epigenetic profiles. Understanding both normal epigenetic development —and how ELS alters maturation of the epigenome— will lay the foundation for targeted interventions to prevent changes in epigenetic development that contribute to neuropsychiatric disease risk.
